# A LiAlO_2_/nitrogen-doped hollow carbon spheres (NdHCSs) modified separator for advanced lithium–sulfur batteries†

**DOI:** 10.1039/c7ra10367k

**Published:** 2018-01-05

**Authors:** Fanqun Li, Furong Qin, Guanchao Wang, Kai Zhang, Peng Wang, Zhian Zhang, Yanqing Lai

**Affiliations:** School of Metallurgy and Environment, Central South University Changsha Hunan 410083 China laiyanqingcsu@163.com

## Abstract

Lithium–sulfur (Li–S) batteries have gained significant attention due to their ultrahigh theoretical specific capacity and energy density. However, their practical commercialization is still facing many intractable problems, of which the most difficult is the shuttle effect of dissolved polysulfides. To restrict the shuttle of polysulfides, herein, a novel double-layer lithium aluminate/nitrogen-doped hollow carbon sphere (LiAlO_2_/NdHCSs)-modified separator was designed. The upper NdHCSs layer on the separator works as the first barrier to physically and chemically adsorb polysulfides, whereas the bottom LiAlO_2_ layer acts as the second barrier to physically block the polysulfides without restricting the Li^+^ transport due to the high ionic conductivity of LiAlO_2_. Cells with the LiAlO_2_/NdHCSs-modified separator showed an initial discharge capacity of 1500 mA h g^−1^ at 0.2C, and a discharge capacity of 543.3 mA h g^−1^ was obtained after 500 cycles at 2C. Especially, when the areal density of the active material was increased to 4.5 mg cm^−2^, the cells retained a discharge capacity of 538.6 mA h g^−1^ after 100 cycles at 0.5C. The outstanding electrochemical performance of Li–S cells with the LiAlO_2_/NdHCSs-modified separators show a new approach for the applications of Li–S batteries.

## Abstract 2

LiAlO_2_/nitrogen-doped hollow carbon spheres (NdHCSs)-modified separators restrict the shuttle of the polysulfides.

## Graphical abstract

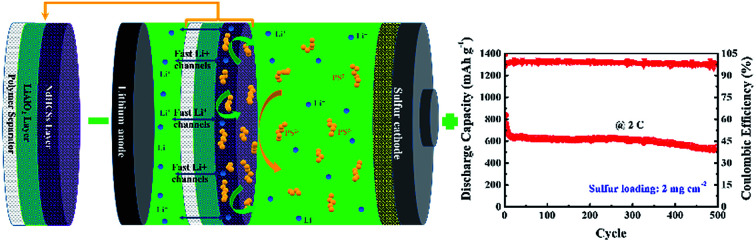

## 1. Introduction

The scarcity of fossil fuels and the ever-growing environmental problems promote the development of renewable energy technologies such as fuel cells, metal–air batteries, and other secondary batteries.^1,2^ Lithium–sulfur (Li–S) batteries have been regarded as one of the most promising candidates for the next-generation energy storage systems. The theoretical specific capacity of sulfur is as high as 1675 mA h g^−1^, and the theoretical energy density of a Li–S battery based on a Li anode and a S cathode is ∼2600 W h kg^−1^.^3–6^ Moreover, the environmental benignity and natural abundance of the elemental sulfur make Li–S batteries more attractive to both the academic and industrial communities.^7,8^ It was reported that the practical energy density of packaged Li–S batteries could be 400–600 W h kg^−1^, which was much higher than that of the commercial LiCoO_2_/C batteries.^9–11^ However, there are some intractable issues, such as low utilization of sulfur, poor cycle stability, and dissolution of the intermediates formed in the discharge–charge processes, hindering the practical applications of Li–S batteries. It is recognized that sulfur and its final discharge products (Li_2_S_2_ or Li_2_S) show ultralow ionic and electronic conductivity, which in turn is responsible for the low utilization of active materials.^12,13^ Dissolution of the intermediates (Li_2_S_*x*_, 3 ≤ *x* ≤ 8) not only causes a continuous loss of active materials, but also results in shuttle phenomenon that leads to a low coulombic efficiency.^14–16^ In addition, huge volume expansion (∼80%) occurs during discharge processes due to the different volume densities of S and Li_2_S, which would results in an unstable structure of the electrode.^17,18^

In the past few years, many efforts have been dedicated towards improving the electrochemical performance of Li–S batteries mainly through designing various porous architectures to confine sulfur. Hosts for accommodating insulating sulfur include carbon materials,^19–22^ polymers,^23–25^ inorganic oxides,^7,8,26^ and other organic compounds.^27^ These materials have shown remarkable improvement in the performance of Li–S batteries because they can provide high electrical conductivity for the sulfur cathode and confine the active materials within the porous structure with physical or chemical entrapment, thus restricting the shuttle of polysulfides (PS). However, for better performance, sulfur needs to be well dispersed within the pores of the host material. This involves complex fabrication processes, which in turn makes sulfur inconvenient to be used in practical application for Li–S batteries.^28–30^

Construction of a new cell configuration to restrict the shuttle of polysulfides is demonstrated to be another effective way to improve the electrochemical performance of lithium–sulfur batteries. This involves insertion of interlayers with high physical or chemical adsorbability between the conventional sulfur cathode and the separator^14,31–33^ and introduction of adsorption layers on the top surface of the conventional sulfur cathode (cathode modification),^34^ or on the side of the separator facing the sulfur cathode (separator modification).^28,30,35^ These layers not only restrict the shuttle of polysulfides but also provide extra active sites for the deposits of Li_2_S_2_ or Li_2_S. With these new configurations, conventional sulfur cathodes that fabricated by simple mixing could be directly used for the cell-assembly, avoiding complicated procedures for preparing sulfur-based composites. Specifically, separator modification is more achievable since a separator possesses good mechanical ability and flexibility. Many materials, including carbon materials,^28^ polymers,^36,37^ metal oxides,^38–40^ metal sulphides,^41^ and hybrid materials,^29,30^ with different functionalities have been applied to construct layers on the separators. With these modified separators, Li–S batteries have shown excellent electrochemical performances. Although these layers can physically or chemically entrap the dissolved polysulfides, some of them can still penetrate the separator. Lately, Bai *et al.* used a metal–organic framework (MOF) to modify the separator for lithium–sulfur batteries.^42^ The MOF was used as an ionic sieve to restrict the shuttle of S_*x*_^2−^ anions, which resulted in an ultra-long cycle life.

Inspired by this, herein, we proposed the use of the ionic conductor LiAlO_2_ and nitrogen-doped hollow carbon spheres (NdHCSs) to construct double layers on the commercial polymer separator to significantly minimize the shuttle of polysulfides. The NdHCSs on the upper layer serve as a secondary current collector and the first absorption layer, whereas LiAlO_2_ at the bottom layer blocks the pores of the separator to restrict the penetration of polysulfides. Since LiAlO_2_ possesses excellent lithium ion conductivity (up to 3 × 10^5^ Ω^−1^ cm^−1^),^43,44^ the transport of lithium ions is not affected. With the LiAlO_2_/NdHCSs-modified separator, the initial discharge capacity is increased from 1013.7 to 1500 mA h g^−1^ at 0.2C. Even at a high current density of 2C, the cells retained a discharge capacity of 543.3 mA h g^−1^ after 500 cycles. In addition, when the areal sulfur loading was increased to 3.0 and 4.5 mg cm^−2^, the Li–S cells demonstrated the discharge capacities of 640 and 538.6 mA h g^−1^ after 100 cycles at 0.5C, respectively.

## 2. Experimental

### 2.1 Material preparation

NdHCSs were obtained using the same synthetic protocols mentioned in our earlier reports.^45^ Typically, silica spheres (2 g, Sigma-Aldrich, 140 nm) were used as the templates and mixed with dopamine (2 g, Sigma-Aldrich) in a Tris-buffer solution (250 mL, pH 8.5) for polymerization under vigorous stirring for 24 h. The polydopamine/SiO_2_ composite was obtained by filtration and dried at 80 °C. Thereafter, it was carbonized under an argon flow at 400 °C for two hours (heating rate was 1 °C min^−1^), followed by another three hours at 800 °C (heating rate was 5 °C min^−1^). After natural cooling, the sample was washed in a HF aqueous solution (15 wt%) for 24 h. For the preparation of LiAlO_2_, Al(NO_3_)_3_·9H_2_O (1.42 g) was mixed with LiOH·H_2_O (0.32 g) in distilled water (200 mL). Then, an appropriate amount of ammonia was added to the solution under magnetic stirring to form a gel. After drying, the resultant product was transferred into a tube furnace and calcinated in air at 500 °C for 4 h (heating rate was 5 °C min^−1^). After washing with deionized water and drying, pure LiAlO_2_ was obtained.

The LiAlO_2_/NdHCSs-modified separator was prepared by a two-step casting procedure. At first, LiAlO_2_ (90 wt%) was mixed with PVDF (10 wt%) using *N*-methyl-2-pyrrolidone (NMP) as the dispersant to make a slurry, which was then coated on a piece of polymer separator (Celgard 2325) *via* the doctor-blade technique. After drying at 60 °C, the slurry composed of NdHCSs/PVDF (9 : 1) was applied on the top of the LiAlO_2_ layer *via* the same procedure. Then, the separator was shaped into a circular membrane with a diameter of 19 mm after drying.

### 2.2 Material characterization

Field emission scanning electron microscopy (SEM, Nova Nano SEM 230 or MIRA 3 LMU) was employed to characterize the morphology. Elements on the surface of the sample were identified by energy-dispersive X-ray spectroscopy (EDX). X-ray diffraction (XRD) measurements were performed using Rigaku 3014 (Cu-Kα). The size distribution of NdHCSs was obtained by the Nano Measurer software.

### 2.3 Electrochemical measurements

A sulfur cathode was prepared by mixing elemental sulfur (70 wt%), carbon black (Super P, 20 wt%), and PVDF (10 wt%) to make a slurry with NMP as the dispersant. Then, this slurry was casted onto a piece of aluminium foil with doctor-blade and then dried at 50 °C in a vacuum oven for 24 h. Then, the electrode was shaped into a circular disk with a diameter of 13 mm. The sulfur loading of sulfur cathodes was tailored by controlling the thickness of the slurry. Li–S cells were assembled in an argon filled glove box. A lithium foil was used as the anode. The electrolyte consisted of 1 M lithium bis(trifluoromethanesulfonyl)imide (LiTFSI) and 0.2 M LiNO_3_ in a mixture of DOL/DME (1 : 1, v/v). For the assembly, 30 mL g_sulfur_^−1^ of electrolyte was added to each cell. Cyclic voltammetry (CV) measurement was performed using a multichannel electrochemical test system (1470E/1400A, Solartron) in the voltage range of 2.8–1.8 V at a scanning rate of 0.2 mV s^−1^.

## 3. Results and discussion

**Fig. 1:**
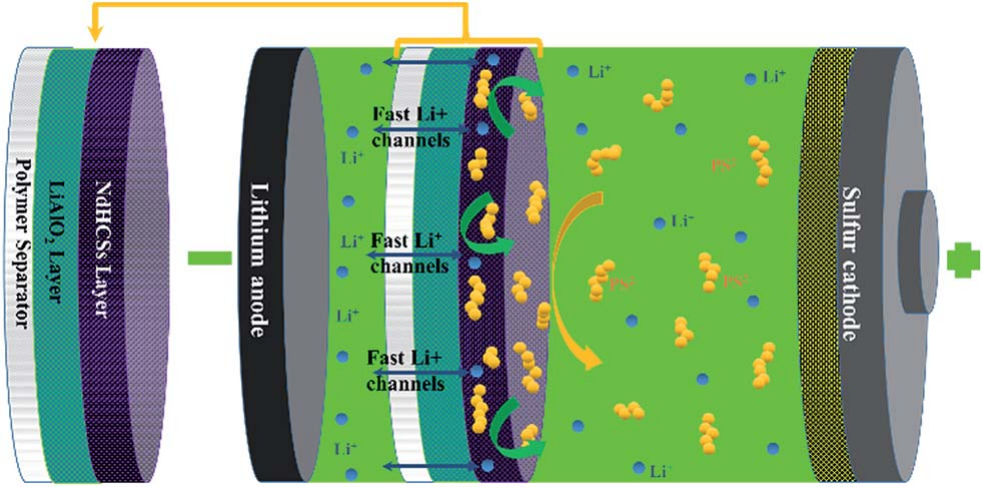
Schematic of the LiAlO_2_/NdHCSs-modified separator and the corresponding Li–S cell configuration.

**Fig. 2:**
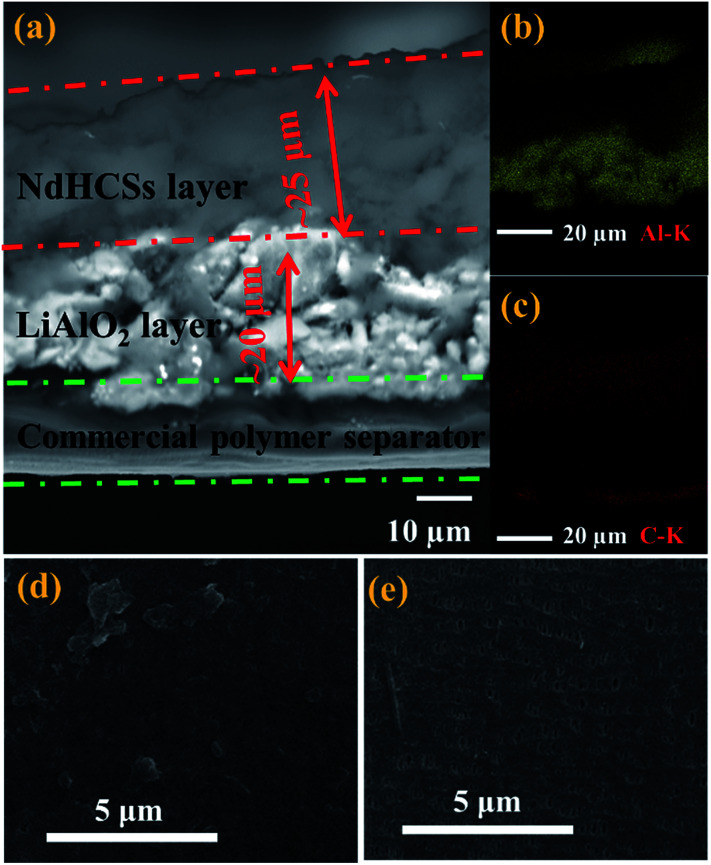
SEM image (a) (cross-section view) of the LiAlO_2_/NdHCSs-modified separator; (b) Al and (c) C element mapping from the EDS analysis of the LiAlO_2_/NdHCSs-modified separator; top-view SEM images of (d) the LiAlO_2_/NdHCSs-modified separator and (e) the unmodified commercial separator. (d) Top view of the side without layers.

The schematic of the LiAlO_2_/NdHCSs-modified separator and the corresponding Li–S cell configuration are presented in Fig. 1. The LiAlO_2_/NdHCSs-modified separator possesses a sandwich structure where LiAlO_2_ layer is located between the commercial polymer separator and the NdHCSs layer. When the dissolution of polysulfides occur, the shuttle of polysulfides is partially adsorbed by the NdHCSs layer with a strong physical and chemical adsorbability. The rest of the polysulfides penetrating the NdHCSs layer are blocked by the LiAlO_2_ layer. Therefore, the loss of active materials is minimized; this results in higher utilization of sulfur and higher coulombic efficiency. The morphology of the LiAlO_2_/NdHCSs-modified separator was investigated first *via* SEM. As shown in the SEM image presented in Fig. 2(a), the sandwich structure of the modified separator is well observed, which demonstrates that the LiAlO_2_/NdHCSs-modified separator is successfully fabricated. EDS mapping results (Fig. 2(b and c)) further demonstrate the interfaces of the separator-LiAlO_2_ and LiAlO_2_–NdHCSs. To demonstrate that the pores of the modified separator are blocked by the LiAlO_2_ layer, the top view SEM image of the modified separator (obtained from the side without layers) is presented in Fig. 2(d), from which it can be clearly seen that the pores are not observed, unlike the SEM image of the unmodified separator displayed in Fig. 2(e).

**Fig. 3:**
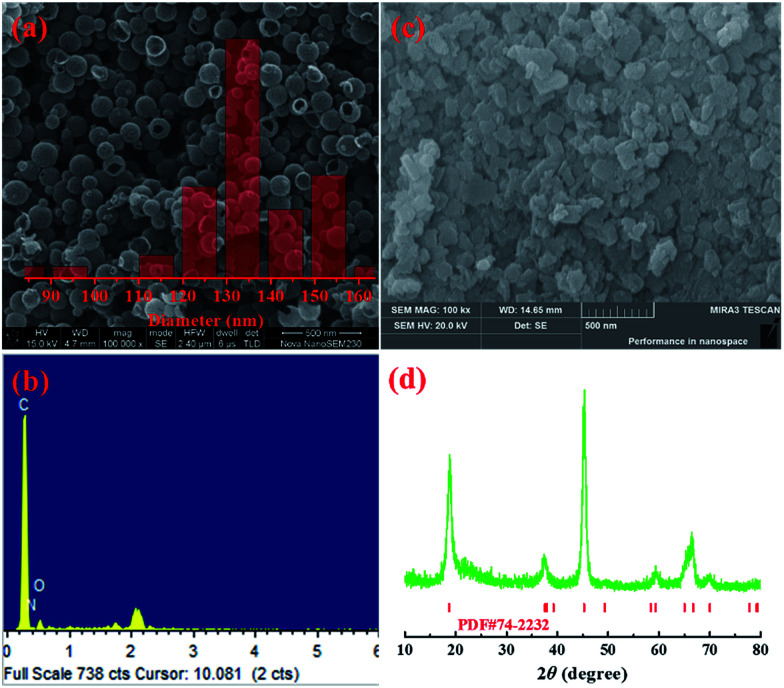
(a) SEM image and size distribution (inset) of NdHCSs and (b) the corresponding EDX measurement results; (c) SEM image of LiAlO_2_ and (d) the XRD pattern.

Fig. 3(a) shows the SEM image and the size distribution of the NdHCSs. It can be observed that the NdHCSs are about 140 nm in diameter, and their hollow structure is well preserved. A signal for the nitrogen element was detected by energy dispersive X-ray (EDX), as presented in Fig. 3(b), which demonstrates the existence of nitrogen element doped in NdHCSs. Further characterization of NdHCSs can be found in our previous study.^45^ Many studies have shown that the nitrogen element can increase electronic conductivity and enhance the chemical adsorbability of polysulfides.^46–48^ Therefore, the NdHCSs layer can possess strong absorption ability for polysulfides, thus improving the electrochemical performance of Li–S cells. Morphology of LiAlO_2_ was also characterized. As shown in the SEM image presented in Fig. 3(c), LiAlO_2_ shows irregular shapes. Fig. 3(d) displays the XRD pattern of the as-prepared LiAlO_2_, which is in line with the α-LiAlO_2_ crystal (JCPDS no. 74-2232), demonstrating that α-LiAlO_2_ is successfully synthesized. LiAlO_2_ has been successfully coated on the surfaces of cathode materials of lithium-ion batteries, which can supply excellent lithium ion conductivity and increase the cycle stability and rate capability.^44,49^ Therefore, the introduced LiAlO_2_ is anticipated to not only absorb and block the polysulfides, but also promote the lithium-ion conductivity and increase the electrochemical performance.

**Fig. 4:**
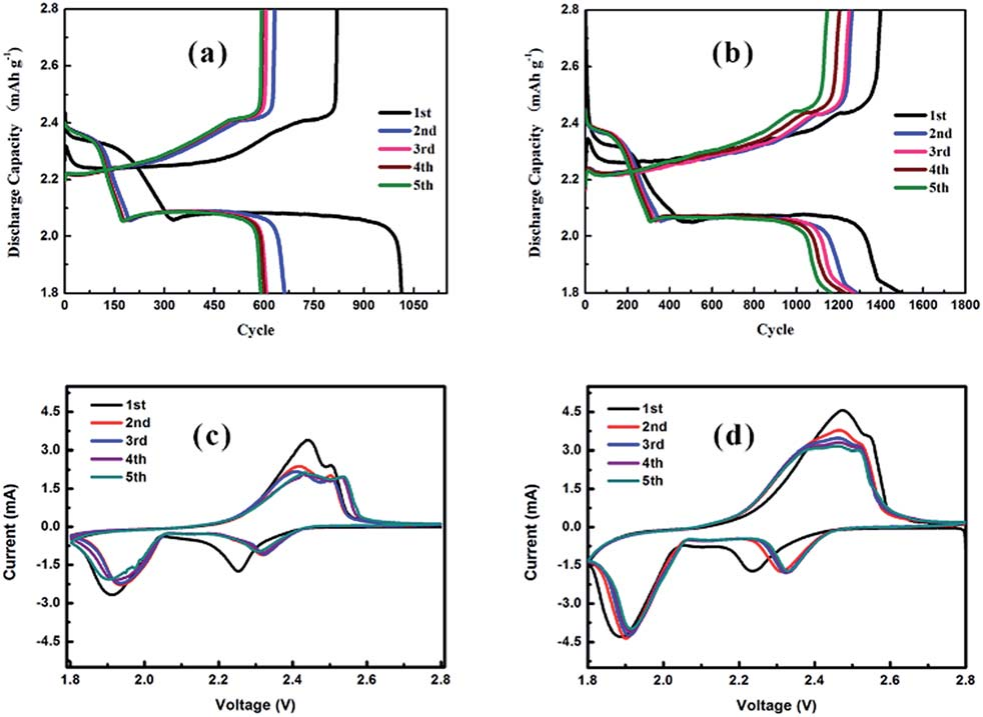
The discharge/charge curves (at 0.2C) of Li–S cells (a) with pristine separator and (b) LiAlO_2_/NdHCSs-modified separator (b). CV curves of Li–S cells (c) with a pristine separator and (d) LiAlO_2_/NdHCSs-modified separator. The sulfur loading of the tested cathodes is ∼2 mg cm^−2^.

The discharge/charge curves and the cyclic voltammograms (CV) of Li–S cells with a pristine separator and modified separator are shown in Fig. 4. As illustrated in Fig. 4(a and b), both cells show two discharge plateaus at ∼2.35 and 2.05 V, which correspond to the conversion of S_8_ to soluble polysulfides (Li_2_S_*n*_, 4 ≤ *n* ≤ 8) and their further reduction to insoluble Li_2_S/Li_2_S_2_, respectively. Moreover, two charge plateaus at ∼2.25 and 2.40 V are displayed, which correspond to the reverse reaction process, namely Li_2_S/Li_2_S_2_ to soluble polysulfides (Li_2_S_*n*_, 4 ≤ *n* ≤ 8) and polysulfides to S_8_, respectively.^50,51^ In addition, the initial discharge capacities of Li–S cells with pristine and LiAlO_2_/NdHCSs-modified separators are 1013.7 and 1500 mA h g^−1^ at a current density of 0.2C, respectively, and the latter indicates longer discharge plateaus and higher discharge capacities in the following discharge/charge cycles. The subsequent CV test results are in good agreement with the discharge/charge curves, as shown in Fig. 4(c and d). It is observed that the curves of the cell with the LiAlO_2_/NdHCSs-modified separator present better overlapping, which indicates that the LiAlO_2_/NdHCSs-modified separator can enhance the stability of the Li–S cell. Since the electrode parameters, such as the areal sulfur loading, are similar, the larger peak area also reflects the higher utilization of sulfur in the Li–S cell with LiAlO_2_/NdHCSs-modified separator.

**Fig. 5:**
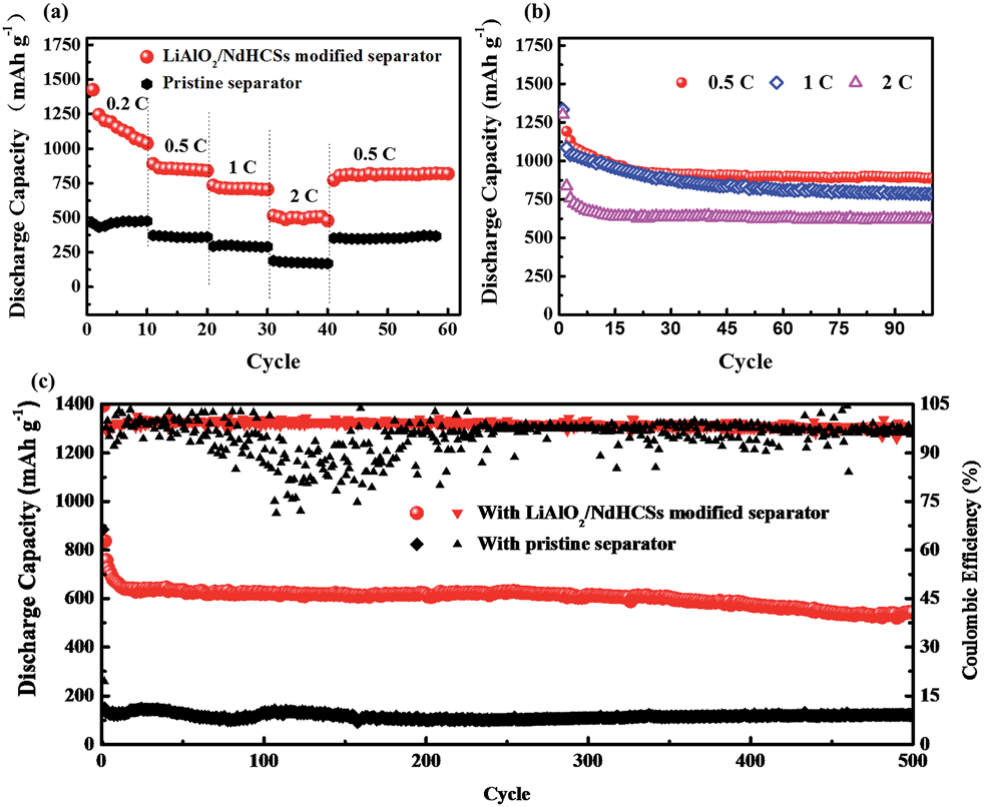
(a) Rate performance of the Li–S cells with the LiAlO_2_/NdHCSs-modified separator and pristine separator. (b) Cycle performance of the Li–S cell with the LiAlO_2_/NdHCSs-modified separator at various rates of 0.5C, 1C, and 2C. (c) Long-term cycle performance of the Li–S cells with the LiAlO_2_/NdHCSs-modified separator and pristine separator at 2C. The sulfur loading of the tested cathodes is ∼2 mg cm^−2^.

Fig. 5(a) shows the rate capabilities of the cells with LiAlO_2_/NdHCSs-modified separator and pristine separator. At 0.5, 1, and 2C, the cell with the LiAlO_2_/NdHCSs-modified separator delivered discharge capacities of 842, 705, and 480 mA h g^−1^, respectively. Comparatively, the cell with a pristine separator barely retained the discharge capacities of 362, 298 and 176 mA h g^−1^. When the rate was changed back to 0.5C, the cell with the LiAlO_2_/NdHCSs-modified separator was able to maintain a discharge capacity of 820 mA h g^−1^, whereas the cell with a pristine separator showed a discharge capacity of 362 mA h g^−1^. Then, the cycle performance of the Li–S cell with LiAlO_2_/NdHCSs-modified separator at different rates was investigated, as shown in Fig. 5(b). It should be pointed out that the first cycle was tested at a low current density of 0.2C. After subsequent 100 cycles at 0.5, 1, and 2C, the cell delivered the discharge capacities of 889.6, 786.2, and 624.8 mA h g^−1^ respectively, exhibiting excellent cycle stability at different rates. To further investigate the electrochemical performance of Li–S cells, a long cycle life test was conducted at a current density of 2C, and the first cycle was tested at a low current density of 0.2C. As shown in Fig. 5(c), after 500 cycles, the discharge capacities of 543.3 mA h g^−1^ and 121 mA h g^−1^ were obtained, indicating excellent cycling stability of the Li–S cell with a LiAlO_2_/NdHCSs-modified separator. In addition, the coulombic efficiency of the cell with a pristine separator showed a huge fluctuation, whereas the cell with a LiAlO_2_/NdHCSs-modified separator exhibited stable coulombic efficiency with an average value as high as 98.8%.

**Fig. 6:**
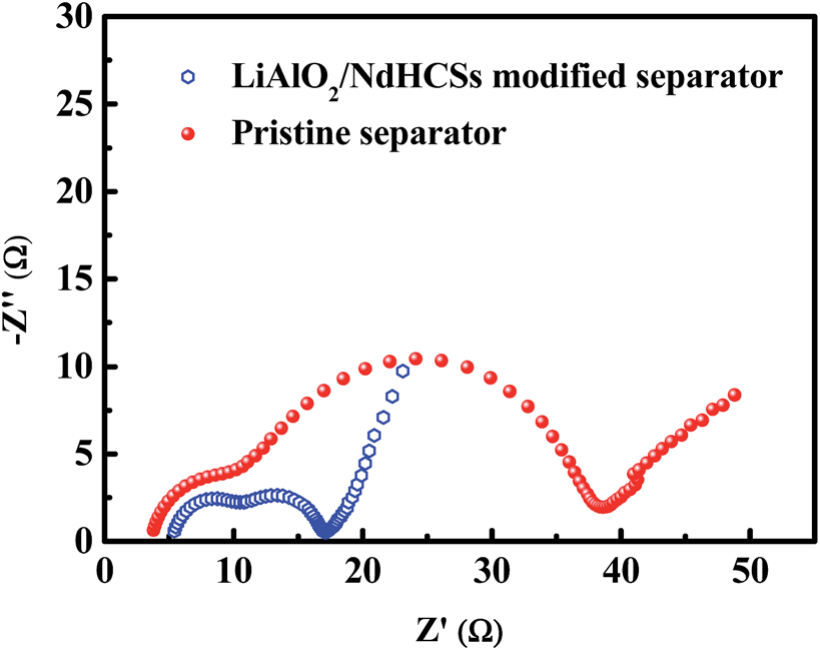
EIS of the cells with the LiAlO_2_/NdHCSs-modified separator and pristine separator.

To demonstrate the faster kinetics in the Li–S cell with the LiAlO_2_/NdHCSs-modified separator, electrochemical impedance spectra (EIS) were obtained after the first cycle. As presented in Fig. 6, the two types of cells show similar Nyquist plots with two semicircles; the first semicircle in the high frequency region is considered to be related to the solid-electrolyte-interface (SEI) film resistance, whereas the second semicircle is related to the charge-transfer resistance (*R*_ct_).^52^ It can be clearly seen that the Li–S cell with a LiAlO_2_/NdHCSs-modified separator shows smaller charge-transfer resistance, which demonstrates that it possesses faster kinetics. This can be one of the reasons for its superior electrochemical performance. To demonstrate the adsorption ability of the NdHCSs to polysulfides, ex situ adsorption measurements were carried out by immersing NdHCSs in a Li_2_S_6_ solution (5 mL, 0.04 mmol L^−1^). Then, the color change was determined by a camera. As presented in the images shown in Fig. S1 (ESI†), the solution with the NdHCSs powder clearly faded after 72 h. To demonstrate that LiAlO_2_ could restrict the shuttle of the dissolved polysulfides, the electrochemical performance of a cell with a LiNO_3_-free electrolyte and a LiAlO_2_-modified separator was measured. As presented in Fig S2(a),† the coulombic efficiency is noticeably enhanced with the LiAlO_2_-modified separator, which demonstrates that LiAlO_2_ can effectively restrict the shuttle effect. The LiAlO_2_/NdHCSs-modified separator further enhances the coulombic efficiency. It also renders a better rate performance, as illustrated in Fig. S2(b).†

**Fig. 7:**
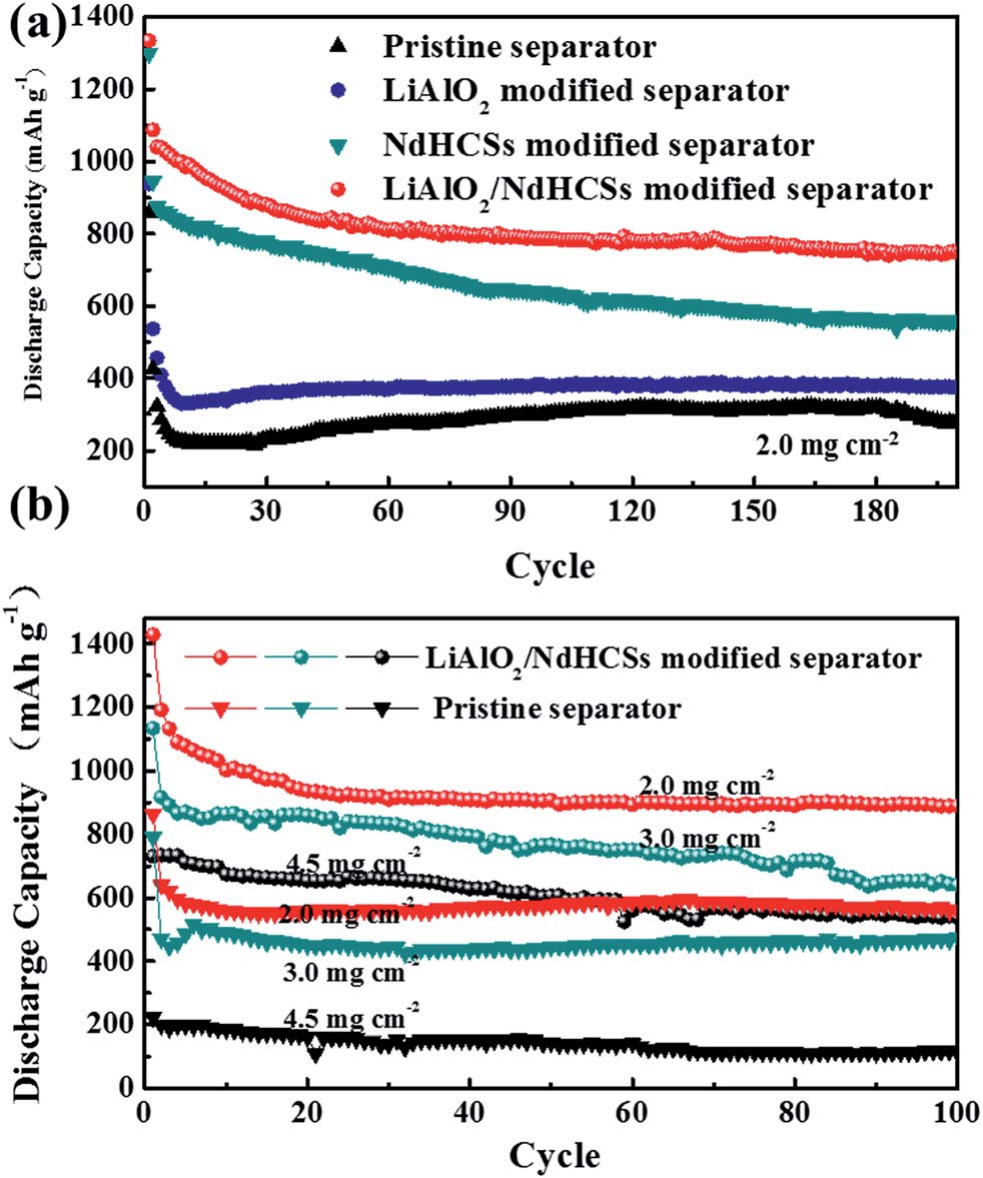
(a) The cycle performance of Li–S cells (sulfur loading = ∼2.0 mg cm^−2^) with the pristine separator, LiAlO_2_-modified separator, NdHCSs-modified separator, and LiAlO_2_/NdHCSs-modified separator at 1C; (b) the cycle performance of Li–S cells with the pristine separator and LiAlO_2_/NdHCSs-modified separator with different areal densities (2.0, 3.0, and 4.5 mg cm^−2^) of active materials.

Fig. 7(a) shows the cycle performance of Li–S cells with different separators. The first cycle was tested at a low current density of 0.2C, and, the following cycles were conducted at 1C. Li–S cells with the pristine separator, LiAlO_2_-modified separator, NdHCSs-modified separator, and LiAlO_2_/NdHCSs-modified separator revealed an increment of initial discharge capacities in sequence. Moreover, the cells respectively maintained the discharge capacities of 276.1, 376.3, 559.4, and 749 mA h g^−1^ after 200 cycles, and the capacity retention rates were 32.2%, 40.1%, 43.1%, and 56.2%, respectively. It can be concluded that the LiAlO_2_/NdHCSs-modified separator can effectively improve the cycle stability of Li–S cells, and the utilization rate of the active material is significantly enhanced. To further investigate the influence of the LiAlO_2_/NdHCSs-modified separator on Li–S cells, cells with different areal densities of active materials were assembled and tested, as shown in Fig. 7(b). The first cycle was tested at a low current density of 0.2C, and the following cycles were conducted at 0.5C. We found that the initial discharge capacities of Li–S cells decreased with the increasing areal densities. When the areal sulfur loading was 2.0, 3.0, and 4.5 mg cm^−2^, Li–S cells with pristine separators maintained the discharge capacities of 560.3, 475.2, and 114.1 mA h g^−1^ after 100 cycles, whereas the cells with LiAlO_2_/NdHCSs-modified separators showed the great discharge capacities of 889.6, 640, and 538.6 mA h g^−1^.

The results demonstrate that the LiAlO_2_/NdHCSs-modified separator can greatly improve the energy density of Li–S cells. The LiAlO_2_ bottom layer on the separator not only blocks the dissolved polysulfides to restrict their shuttling, but also provides fast ionic channels for the transfer Li^+^ ions. Moreover, the upper NdHCSs layer not only works as a second current collector to provide more active sites for the redox reaction, but also restricts the shuttle of polysulfides. As a result, the LiAlO_2_/NdHCSs-modified separator shows great potential for prospective applications of Li–S batteries.

## 4. Conclusions

In conclusion, a novel double-layer LiAlO_2_/NdHCSs-modified separator was prepared to improve the poor cycle stability and fast capacity fading of Li–S cells. The modified separator exhibits significant inhibition of shuttle effect and high utilization of active materials. With the LiAlO_2_/NdHCSs-modified separator, a high initial discharge capacity of 1500 mA h g^−1^ was achieved at 0.2C, and a discharge capacity of 543.3 mA h g^−1^ was obtained after 500 cycles at 2C. Especially, when the areal density of the active material was increased to 4.5 mg cm^−2^, the cells retained a discharge capacity of 538.6 mA h g^−1^ after 100 cycles at 0.5C. The NdHCSs make direct contacts to the S cathode while serving as an upper collector and the first absorption layer. Moreover, LiAlO_2_ can offer more Li^+^ transportation channels and serves as the second absorption layer. Therefore, the double-layer LiAlO_2_/NdHCSs-modified separator shows great potential for further practical applications of Li–S batteries.

## Conflicts of interest

There are no conflicts of interest to declare.

## Supplementary Material

RA-008-C7RA10367K-s001
