# Supplementary material for: A LiAlO2/nitrogen-doped hollow carbon spheres (NdHCSs) modified separator for advanced lithium–sulfur batteries
Source: RSC Adv. 2018 Jan 5;8(3):1632–7. doi: 10.1039/c7ra10367k (PMC9077093; doi:10.1039/c7ra10367k)
Supplement: RA-008-C7RA10367K-s001 [file RA-008-C7RA10367K-s001.pdf]

Electronic Supplementary Material (ESI) for RSC Advances.
This journal is © The Royal Society of Chemistry 2017

Electronic Supplementary Information

$LiAlO_2$/Nitrogen-doped Hollow Carbon Spheres (NdHCSs) Modified Separator for Advanced Lithium-Sulfur Batteries †

Fanqun Li [a], Furong Qin [a], Guanchao Wang [a], Kai Zhang [a], Peng Wang [a], Zhian Zhang [a] and Yanqing Lai [a, *]

[a] School of Metallurgy and Environment, Central South University, Changsha 410083, China

* Corresponding author: laiyanqingcsu@163.com

Supporting Figures

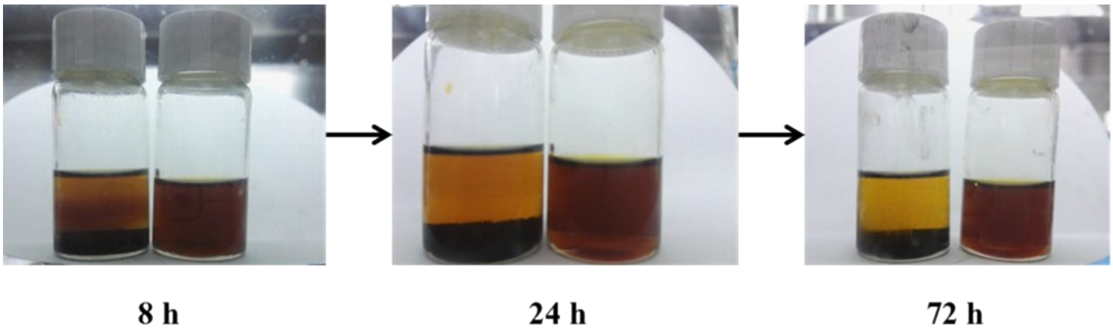

Figure S1. Colour change of the electrolyte with NdHCSs (left) .

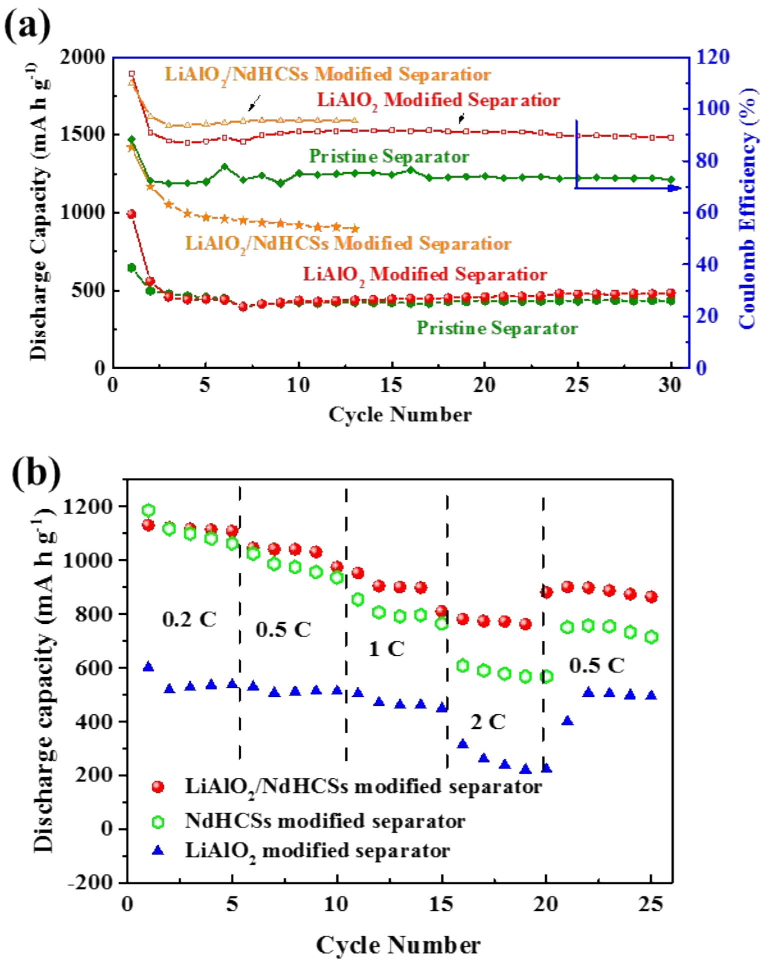

Figure S2. Cycle performance (a) and rate performance (b) of cell with different separator.
